# The Influence of Age on Cancer-Specific and Other-Cause Mortality in Upper Tract Urothelial Carcinoma

**DOI:** 10.3390/cancers18142317

**Published:** 2026-07-18

**Authors:** Roderick Clark, Veenadhari Kollipara, Jay D. Raman

**Affiliations:** Department of Urology, Penn State Milton S Hershey Medical Center, Hershey, PA 17033, USA

**Keywords:** upper tract urothelial carcinoma, age, cancer-specific mortality, competing risk

## Abstract

**Background**: Upper tract urothelial carcinoma (UTUC) is a rare malignancy with prognosis predicated on clinical and pathological factors. Radical nephroureterectomy is the standard treatment for higher risk localized UTUC, although competing mortality may influence optimal treatment selection in older patients. **Methods:** A retrospective population-based cohort study was conducted using the Surveillance, Epidemiology, and End Results program. Individuals diagnosed with UTUC of the renal pelvis or ureter between 2000 and 2017 were included. Patients with unknown tumor grade or size were excluded. **Results:** A total of 10,894 patients were included. During a median follow-up of 26 months, 57% died from any cause and 27% died from UTUC. For each 5-year increase in age of diagnosis, the cancer-specific mortality risk rises more in low-grade patients (21% increased riskjt*p*: 0.008) than high-grade patients (9% increased risk *p*: 0.023). Other-cause mortality risk rose markedly with increasing age (SHR 3.54 for ages 85+, *p* < 0.001). **Conclusions:** Age at diagnosis is significantly associated with cancer-specific mortality in UTUC. Incorporating age and competing risk into risk stratification may help inform shared decision-making regarding treatment approaches in older patients.

## Simple Summary

Upper tract urothelial carcinoma is a rare and aggressive cancer of the kidney and ureter that predominantly affects older individuals. Current guidelines recommend surgical removal of the affected kidney and ureter as the standard treatment, but this operation carries significant risks, particularly in elderly patients who may be more likely to die from other health conditions before the cancer itself causes death. Using a large national cancer registry of nearly 11,000 patients, we examined how age at diagnosis influences the risk of dying from the cancer versus other causes, and whether tumor aggressiveness modifies this relationship. Our findings suggest that age should be incorporated into treatment decision-making for this disease, particularly when counseling older patients about the risks and benefits of aggressive surgical treatment.

## 1. Introduction

Upper tract urothelial carcinoma (UTUC) is a rare but aggressive malignancy arising from the urothelial lining of the renal pelvis or ureter. The disease has an estimated incidence of 1–2 cases per 100,000 individuals annually and disproportionately affects older adults, with a male predominance [1,2]. Despite representing only 5–10% of all urothelial carcinomas, UTUC carries a substantially worse prognosis than bladder cancer, with a 5-year cancer-specific survival rate below 50% for muscle-invasive disease [3]. Radical nephroureterectomy (RNU) with ipsilateral bladder cuff excision is the established standard of care for high-risk localized UTUC [4,5]. However, RNU is associated with significant perioperative morbidity, long-term renal function impairment, and increased cardiovascular risk—consequences that may be disproportionately harmful in older patients with pre-existing comorbidities [6,7].

Current risk stratification frameworks for UTUC focus almost exclusively on tumor-level factors, including grade, stage, location, multifocality, and imaging characteristics [4,5]. Patient-level factors such as age, frailty, functional status, and comorbidity burden are notably absent from guideline-endorsed decision pathways, despite their well-established relevance to treatment tolerance and non-cancer mortality risk. This represents a clinically significant gap, particularly given the predominantly older demographic of patients with UTUC.

The concept of competing risks is well established in oncology. In settings where patients face a high likelihood of dying from causes other than their cancer, aggressive cancer-directed treatment may confer limited survival benefit while exposing patients to substantial procedural harm. Competing risk analyses are particularly relevant in malignancies of older adults. For example, the relationship between age and competing mortality has been well characterized in prostate cancer, where older patients are increasingly more likely to die from comorbid conditions than from the cancer itself [8,9]. Similar patterns have been described in breast cancer and other solid tumors [10,11]. Whether analogous age-dependent competing risk dynamics exist in UTUC has not been systematically examined.

A small number of population-based studies have examined age-related outcomes in UTUC. Xylinas and colleagues reported that older age was independently associated with worse overall survival following RNU, but did not explicitly differentiate cancer-specific from non-cancer mortality [12]. Similarly, Chromecki and colleagues demonstrated that incidence and disease-specific survival varied by age in UTUC, though competing risk methodology was not applied [13]. More recent registry-based analyses have begun to address this gap, but have not examined the interaction between age and tumor grade on cause-specific outcomes [14,15].

Several studies have investigated the role of endoscopic management as an alternative to RNU in selected UTUC patients, particularly those with low-risk disease or imperative indications [16,17,18]. Seisen and colleagues reported that endoscopic management may achieve comparable cancer-specific outcomes in carefully selected elderly patients, suggesting that treatment de-escalation in older adults with high competing mortality risk is clinically feasible [16]. However, the identification of which patients stand to benefit from less aggressive management has remained challenging in the absence of patient-level competing risk data stratified by both age and tumor grade.

The objective of this study was to characterize the association between age at diagnosis and both cancer-specific and other-cause mortality in a large, population-based cohort of UTUC patients, and to determine whether tumor grade modifies this age-dependent relationship. We hypothesized that the relative contribution of cancer-specific versus other-cause mortality would shift substantially with advancing age, and that this shift would be more pronounced in low-grade than high-grade disease; these findings could inform more individualized treatment discussions for older patients.

## 2. Materials and Methods

### 2.1. Study Design, Setting, and Data Source

We conducted a population-based retrospective cohort study using data from the Surveillance, Epidemiology, and End Results (SEER) program [19].

### 2.2. Population

Individuals diagnosed with urothelial carcinoma of the renal pelvis or ureter between 2000 and 2017 were included. Patients with unknown grade or size of the tumor were excluded. Patients were divided into 6 age categories: <65, 65–69, 70–74, 75–79, 80–84, and 85+. A total of 10,894 patients were included. Data about the treatment course for patients was not available.

### 2.3. Outcomes, Exposure and Covariates

The primary outcome was mortality rates of individuals with UTUC, based on age category. Death from other causes was the competing event.

### 2.4. Statistical Analysis

The Aalen–Johansen estimator was used to generate cumulative incidence functions, accounting for competing events [20]; Fine and Gray proportional subdistribution hazard models were used for regression analyses [21] and all effect estimates are reported as SHRs with 95% confidence intervals. Statistical significance was defined as *p* < 0.05 and interaction term significance was assessed using the joint Wald chi-squared test. Age effect was modeled in 5-year increments and separate analyses were performed for low- and high-grade disease.

Analyses were performed using Stata version 19. These analyses were performed using publicly available data and thus did not require ethics approval.

## 3. Results

**Figure 1:**
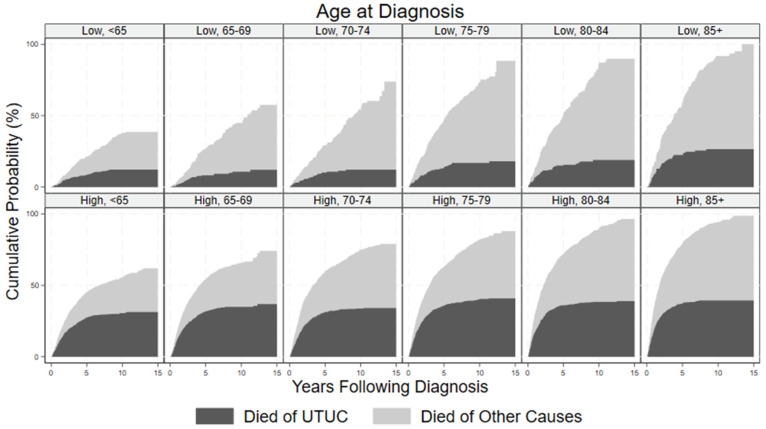
Percentage of patients who died of UTUC versus other causes, stratified by age.

We identified 10,894 individuals diagnosed with UTUC over the study period. Of these patients, 18% (1916) were diagnosed with low-grade tumors, and 82% (8978) were diagnosed with high-grade tumors (Table 1). The median follow-up was 26 months (IQR = 6 to 28 months). During the post-follow-up period, 57% of participants died of any cause, while 27% died of UTUC. The median survival for those who died of all causes was 19 months (IQR = 8 to 41 months). The median survival for those who died of UTUC was 14 months (IQR = 6 to 28 months) (Table 2 and Figure 1).

**Table 1:** Distribution of patients by tumor grade and age group (N = 10,894).

Age Group	Low Grade n (%)	High Grade n (%)	Total n (%)
<65	545 (28.4%)	1984 (22.1%)	2529 (23.2%)
65–69	293 (15.3%)	1288 (14.4%)	1581 (14.5%)
70–74	292 (15.2%)	1521 (16.9%)	1813 (16.6%)
75–79	334 (17.4%)	1657 (18.5%)	1991 (18.3%)
80–84	258 (13.5%)	1466 (16.3%)	1724 (15.8%)
85+	194 (10.1%)	1062 (11.8%)	1256 (11.5%)
Total	1916 (100%)	8978 (100%)	10,894 (100%)
**Events**			
UTUC death	247	2673	2920 (26.8%)
Other-cause death	638	2587	3225 (29.6%)
Censored	1031	3718	4749 (43.6%)

Percentages represent row proportions within each grade group. UTUC = upper tract urothelial carcinoma.

**Table 2:** Cancer-specific death by tumor grade and age group.

	Low Grade (n = 1916)		High Grade (n = 8978)	
Age Group	5-Year CIF (95% CI)	10-Year CIF (95% CI)	5-Year CIF (95% CI)	10-Year CIF (95% CI)
<65	8.7% (5.0–12.5%)	25.8% (21.3–30.3%)	17.9% (13.3–22.4%)	25.0% (20.9–29.2%)
65–69	19.1% (14.1–24.2%)	34.1% (20.1–48.1%)	25.1% (20.1–30.2%)	30.9% (27.7–34.2%)
70–74	19.0% (12.9–25.0%)	42.1% (34.2–50.0%)	29.5% (26.9–32.2%)	41.3% (37.6–44.9%)
75–79	20.8% (7.8–33.8%)	56.7% (50.1–63.2%)	32.6% (28.1–37.1%)	40.5% (37.4–43.6%)
80–84	31.4% (20.3–42.6%)	68.1% (60.4–75.9%)	36.2% (34.1–38.3%)	51.1% (47.8–54.3%)
85+	37.0% (29.3–44.6%)	65.2% (55.3–75.1%)	39.4% (36.4–42.4%)	55.0% (51.4–58.6%)

CIF = cumulative incidence function estimated by the Aalen–Johansen estimator; accounts for other-cause death as a competing event. 95% CIs derived by normal approximation from 1000 bootstrap replications. CIF values expressed as percentages.

The total cohort was then stratified based on age [Table 2]. Age of diagnosis categories were set as: <65, 65–69, 70–74, 75–79, 80–84, and 85+. At the 5-year follow-up, younger patients (<65) have double the cancer-specific mortality risk for high-grade (18%) vs. low-grade (9%) disease. For older individuals (85+), the cancer-specific mortality risk is similar for high-grade (40%) vs. low-grade (37%) disease. At 10-years, older patients have higher cancer-specific risk for low-grade, but not high-grade disease. The age trend in cancer-specific SHR is steeper in low-grade than high-grade disease. In low-grade disease, the 10-year cancer-specific risk nearly triples by age of diagnosis from 26% (<65 y/o) to 69% (80–84 y/o). For high-grade disease this difference is less: 25% (<65 y/o) to 51% (80–84 y/o). The number of patients remaining at risk declined substantially over time, particularly beyond 5 years, reflecting high early competing mortality in this cohort.

We then performed multivariate analysis for cancer-specific mortality risk. High-grade disease is associated with significant increased risk (SHR: 2.94 (95% CI: 2.23–3.86) *p* < 0.01) among younger individuals but the interaction term indicates the magnitude of this effect lessens with older age at diagnosis (Table 3). For low-grade disease, the age trend becomes significant when diagnosed in older individuals: 75–79 (SHR: 1.55 *p*: 0.02), 80–84 (SHR: 1.67 *p*: 0.012) and 85+ (SHR: 2.56 *p*: <0.001). The interaction between grade and age was significant (joint Wald chi-squared test: 12.91 *p*: 0.02). The risk of cancer-specific death associated with high-grade disease was highest among younger individuals and nearly equal in the oldest individuals.

Disease grade was not associated with other-cause mortality (SHR: 1.19 (95% CI: 0.96–1.48) *p*: 0.107), whereas age was increasingly associated with other-cause mortality risk as expected (65–69 SHR: 1.62 *p*: 0.001 vs. 85+ SHR: 3.54 *p* < 0.01).

We performed linear tests of the age trend which were significant for all groups (*p*: <0.001) (Table 4). The effect of age was higher in low-grade disease for both cancer-specific and other-cause mortality. For each 5-year increase in age of diagnosis, the cancer-specific mortality risk rises more in low-grade patients (21% increased joint Wald chi-squared test *p*: 0.008) than high-grade patients (9% increase joint Wald chi-squared test *p*: 0.023).

## 4. Discussion

In this population-based analysis of 10,894 patients with UTUC, we demonstrate that age at diagnosis is significantly and independently associated with cancer-specific mortality, and that this relationship is modified by tumor grade. The age-dependent gradient in cancer-specific mortality was substantially steeper in low-grade than in high-grade disease, reflecting a differential competing risk dynamic across histological subtypes. Older patients were increasingly more likely to die from non-cancer causes as age advanced, a pattern that was more pronounced in low-grade tumors where the cancer-specific risk gradient was greatest.

### 4.1. Age, Competing Risks, and Cancer-Specific Mortality

Our findings are consistent with well-characterized competing risk phenomena in other urological and oncological malignancies. In prostate cancer, Clark and colleagues demonstrated that the probability of dying from prostate cancer decreases significantly with advancing age, driven by rising non-cancer mortality risk [8]. Analogous patterns have been described in breast cancer and non-melanoma skin cancer [9,10,11]. Importantly, these observations do not imply that UTUC is biologically less aggressive in older patients; rather, competing comorbidities increasingly dominate the overall mortality landscape as age advances. The present study is among the first to formally quantify this dynamic in UTUC using competing risks methodology with grade-specific stratification.

The differential age gradient we observe between low- and high-grade disease warrants careful interpretation. At younger ages, high-grade disease confers substantially higher cancer-specific mortality than low-grade disease as expected, given well-established differences in tumor biology. However, with advancing age, the cancer-specific risk differential narrows considerably, driven by a steeper age effect in low-grade disease. This convergence at older ages likely reflects several factors: the relatively indolent natural history of low-grade UTUC affords more time for competing mortality to accrue; older patients with low-grade tumors may experience greater absolute gains in non-cancer mortality risk relative to their younger counterparts; and survivorship effects may enrich older cohorts with biologically distinct disease phenotypes. These data reinforce the need for individualized risk assessment rather than grade-based stratification alone.

The role of age as an imperfect surrogate for unmeasured competing risk factors deserves specific acknowledgment. Chronological age likely captures a cluster of clinically relevant but unmeasured constructs including comorbidity burden, functional reserve, frailty, polypharmacy, and social support that collectively determine an individual’s vulnerability to non-cancer death. The observed age gradient in other-cause mortality is therefore best understood as a composite reflection of these factors rather than a direct biological effect of age per se [22]. This distinction is clinically meaningful: two 80-year-old patients may have dramatically different competing mortality profiles depending on their functional status and comorbidity burden.

### 4.2. Implications for Risk Stratification and Clinical Decision-Making

Current AUA and EAU guidelines for UTUC stratify risk primarily based on tumor-level factors, including grade, stage, imaging characteristics, and cytology findings [4,5]. While tumor biology is unquestionably a major determinant of oncologic outcomes, our findings suggest that patient-level factors, particularly age, substantially modify the clinical relevance of these tumor characteristics. For example, high-grade UTUC in a young patient carries markedly different prognostic implications than the same histological finding in an 85-year-old, when competing mortality risk is explicitly accounted for.

Incorporating age into UTUC risk stratification does not imply withholding curative therapy based on chronological age alone. Rather, it supports a more nuanced framework in which age serves as a surrogate for life expectancy and competing mortality risk, thereby helping clinicians frame shared decision-making discussions more accurately. An otherwise healthy 60-year-old with high-grade UTUC may derive substantial oncologic benefit from RNU, while an 85-year-old with multiple comorbidities may reasonably prioritize endoscopic management or symptom control even in the presence of adverse tumor characteristics [16,17,18,23].

This approach aligns with emerging paradigms in geriatric oncology that emphasize individualized treatment intensity and competing-risk-informed decision-making, particularly for older adults with solid tumors [22,24,25]. Several studies have evaluated UTUC management in elderly populations: Seisen and colleagues demonstrated comparable cancer-specific survival between RNU and endoscopic management in carefully selected elderly UTUC patients, supporting the clinical viability of de-escalated treatment strategies [16]. Marchioni and colleagues similarly reported acceptable oncologic outcomes with conservative endoscopic management in frail UTUC patients [17]. Our findings extend this literature by providing a formal quantification of how age and grade interact to modulate the competing risk balance.

### 4.3. Comparison with the Prior UTUC Literature

Prior population-based studies of UTUC have largely focused on overall survival and cancer-specific survival without explicitly examining how age modifies cause-of-death patterns. Soria and colleagues provided a comprehensive epidemiological review of UTUC, emphasizing its aggressive nature and predilection for older individuals, but did not address competing mortality risk across age groups [1]. Ishikawa and colleagues demonstrated worse overall survival with increasing age following RNU in a multi-institutional cohort, but the relative contributions of cancer-specific versus non-cancer mortality were not examined [12]. Raman and colleagues described incidence and survival trends using SEER data, providing important epidemiologic context, but again without competing risk stratification [2].

Our study extends this literature by specifically quantifying the proportion of deaths attributable to UTUC versus other causes across age and grade strata, a distinction that is clinically most relevant when counseling patients regarding the trade-offs of aggressive treatment. This distinction is critical in a malignancy with high baseline mortality, where aggressive treatment may not translate into meaningful net survival benefit for all patients, particularly those with substantial competing mortality risk. Notably, our findings are consistent with those of Kaag and colleagues, who demonstrated that post-RNU chronic kidney disease limits access to perioperative chemotherapy and independently increases non-cancer mortality, suggesting that the procedural consequences of RNU itself may amplify competing risk in older patients [6,7].

### 4.4. Strengths and Limitations

A primary strength of this study is the use of a large, population-based dataset that captures real-world outcomes across a diverse national population. The size of the cohort enabled robust stratification across six age categories and precise estimation of cause-specific mortality in a rare malignancy. The application of formal competing risks methodology, including Fine and Gray regression with grade–age interaction testing, provides more clinically interpretable estimates than conventional survival analyses that censor competing events [20,21].

Several important limitations merit consideration. First, SEER does not capture comorbidity burden, functional status, or frailty, all of which are likely to mediate the observed age–mortality relationship. Age therefore serves as an imperfect but pragmatic surrogate for these clinically relevant constructs. Second, detailed treatment data (including receipt of RNU, endoscopic management, systemic chemotherapy, or palliative care) were not available for the study period, precluding assessment of how treatment patterns varied by age and grade and influenced outcomes. Third, cause-of-death classification is subject to misclassification in administrative datasets, although such error would likely attenuate rather than exaggerate observed age-related trends [26]. Fourth, the number of patients at risk at 10 years declines substantially in each stratum; 10-year cumulative incidence estimates should therefore be interpreted cautiously. Finally, because SEER does not capture information on Lynch syndrome status, hereditary contributions to UTUC, which may disproportionately affect younger patients, could not be adjusted for [27].

Despite these limitations, the observed gradient in cancer-specific mortality across age groups is biologically plausible and consistent with competing risk theory and patterns observed in other malignancies. Our results are consistent across multiple analytical approaches and provide the most comprehensive characterization to date of age–grade interactions in UTUC cause-specific mortality.

### 4.5. Future Directions

Future studies should incorporate comorbidity indices (e.g., Charlson Comorbidity Index), frailty measures, and treatment-level data to further refine individualized risk prediction in UTUC. Decision-analytic modeling, incorporating competing mortality risk alongside tumor biology, treatment response probability, and procedural risk could help quantify the net benefit of aggressive treatment across age and comorbidity strata. Integration of genomic and molecular biomarkers may further refine risk stratification beyond grade and stage [27,28]. Ultimately, development and prospective validation of a unified competing risk model that incorporates patient-level factors alongside tumor characteristics represents an important next step toward precision risk stratification in UTUC.

## 5. Conclusions

In this large population-based study, we found that age at diagnosis is significantly associated with both cancer-specific and other-cause mortality in UTUC, and that this relationship is modified by tumor grade. Older patients with UTUC are progressively more likely to die from competing comorbid conditions rather than from their cancer, with this competing risk dynamic more pronounced in low-grade than high-grade disease. These findings highlight the importance of incorporating patient age and competing mortality risk alongside tumor characteristics in UTUC risk stratification frameworks. Individualized treatment discussions, particularly regarding the risks and benefits of radical nephroureterectomy in older patients, should account for the competing risk context described here. Future studies integrating comorbidity data, treatment information, and molecular tumor profiling will be essential to refine and prospectively validate competing-risk-informed treatment algorithms for UTUC.

## Figures and Tables

**Table 3:** Hazard models for cancer-specific and other-cause mortality.

Variable	UTUC-Specific Mortality SHR (95% CI)	*p*-Value	Other-Cause Mortality SHR (95% CI)	*p*-Value
*Tumor Grade*				
High grade (ref: Low grade)	2.94 (2.23–3.86)	<0.001	1.19 (0.96–1.48)	0.107
*Age Group*				
<65 (reference)	1.00 (ref)	—	1.00 (ref)	—
65–69	0.90 (0.57–1.42)	0.657	1.62 (1.23–2.13)	0.001
70–74	1.02 (0.66–1.60)	0.919	1.83 (1.40–2.40)	<0.001
75–79	1.55 (1.06–2.26)	0.022	2.92 (2.30–3.72)	<0.001
80–84	1.67 (1.12–2.50)	0.012	3.26 (2.55–4.17)	<0.001
85+	2.56 (1.74–3.77)	<0.001	3.54 (2.74–4.58)	<0.001
*Grade × Age Interaction*				
High grade × 65–69	1.34 (0.83–2.14)	0.229	0.81 (0.59–1.10)	0.179
High grade × 70–74	1.14 (0.72–1.82)	0.569	0.95 (0.70–1.28)	0.729
High grade × 75–79	0.94 (0.63–1.39)	0.753	0.60 (0.46–0.79)	<0.001
High grade × 80–84	0.87 (0.57–1.32)	0.507	0.74 (0.56–0.98)	0.037
High grade × 85+	0.60 (0.40–0.90)	0.015	0.80 (0.60–1.08)	0.142
Wald chi^2^ (11)	326.80	<0.001	442.74	<0.001
Interaction Wald chi^2^ (5)	12.91	0.024	17.34	0.004
Observations	10,894	—	10,894	—
Events	2920	—	3225	—
Competing events	3225	—	2920	—

SHR = subdistribution hazard ratio from Fine & Gray competing risks regression. Robust (sandwich) standard errors used throughout. Interaction *p*-values from Wald test (likelihood ratio test invalid with robust VCE). Reference categories: low-grade tumor, age < 65 years. UTUC = upper tract urothelial carcinoma.

**Table 4:** Linear age trend tests within each grade group.

Grade	Outcome	SHR per 5-yr Age Increase (95% CI)	*p*-Value	Wald chi^2^ (df)
Low grade	UTUC-specific mortality	1.21 (1.12–1.30)	<0.001	25.55 (df = 1)
Low grade	Other-cause mortality	1.30 (1.25–1.36)	<0.001	152.65 (df = 1)
High grade	UTUC-specific mortality	1.09 (1.07–1.11)	<0.001	58.67 (df = 1)
High grade	Other-cause mortality	1.22 (1.19–1.24)	<0.001	302.76 (df = 1)
***Grade × age interaction (combined model)***				
	UTUC-specific mortality	0.980/yr (0.965–0.995)	0.008	—
	Other-cause mortality	0.990/yr (0.981–0.999)	0.023	—

Age modeled as continuous ordinal score using group midpoints (62, 67, 72, 77, 82, 88 years). SHRs for the grade-specific trend converted to per 5-year age increment for interpretability. Grade × age interaction tests whether the age trend in mortality differs significantly between low- and high-grade tumors (*p*-values from Wald test on the interaction term in the combined model).

## Data Availability

Data for this study is freely available at https://seer.cancer.gov/ (accessed on 7 July 2026).
